# The ubiquitin ligase KBTBD8 regulates PKM1 levels via Erk1/2 and Aurora A to ensure oocyte quality

**DOI:** 10.18632/aging.101802

**Published:** 2019-02-20

**Authors:** Yan-Ru Li, Rui-Rui Peng, Wen-Yi Gao, Peng Liu, Liang-Jian Chen, Xiao-Lan Zhang, Na-Na Zhang, Yang Wang, Lei Du, Feng-Yu Zhu, Li-Li Wang, Cong-Rong Li, Wen-Tao Zeng, Jian-Min Li, Fan Hu, Dong Zhang, Zhi-Xia Yang

**Affiliations:** 1 State Key Lab of Reproductive Medicine, Nanjing Medical University, Nanjing 211166, Jiangsu, P.R. China; 2 Reproductive Medical Center, Huzhou Maternity and Child Health Care Hospital, Huzhou 313000, Zhejiang, P.R. China; 3 Nanjing Maternity and Child Health Care Hospital, Nanjing 210004, Jiangsu, P.R. China; 4 Department of Center for Medical Experiments, Third Xiang-Ya Hospital of Central South University, Changsha 410013, Hunan, P.R. China; 5 Animal Core Facility, Nanjing Medical University, Nanjing 211166, Jiangsu, P.R. China; * Equal contribution

**Keywords:** ubiquitin ligase, KBTBD8, PKM1, Erk1/2, Aurora A, oocyte

## Abstract

Tight control of energy metabolism is essential for normal cell function and organism survival. PKM (pyruvate kinase, muscle) isoforms 1 and 2 originate from alternative splicing of PKM pre-mRNA. They are key enzymes in oxidative phosphorylation and aerobic glycolysis, respectively, and are essential for ATP generation. The PKM1:PKM2 expression ratio changes with development and differentiation, and may also vary under metabolic stress and other conditions. Until now, there have been no reports about the function and regulation of PKM isozymes in oocytes. Here, we demonstrate that PKM1 or PKM2 depletion significantly disrupts ATP levels and mitochondrial integrity, and exacerbates free-radical generation and apoptosis in mouse oocytes. We also show that KBTBD8, a female fertility factor in the KBTBD ubiquitin ligase family, selectively regulates PKM1 levels through a signaling cascade that includes Erk1/2 and Aurora A kinases as intermediates. Finally, using RNA sequencing and protein network analysis, we identify several regulatory proteins that may be govern generation of mature PKM1 mRNA. These results suggest KBTBD8 affects PKM1 levels in oocytes via a KBTBD8→Erk1/2→Aurora A axis, and may also affect other essential processes involved in maintaining oocyte quality.

## Introduction

Proper energy metabolism is essential for optimal cell function. ATP levels decrease with aging, a major risk factor for cardiovascular disease, cancer, diabetes, obesity, and neurodegenerative diseases. Restoring or maintaining normal energy metabolism increases the lifespan of aging animals [1–4]. PKM (pyruvate kinase, muscle) isozymes are crucial for ATP generation. The two major PKM isoforms, PKM1 and PKM2, are generated by alternative splicing of PKM pre-mRNA. The embryonic pyruvate kinase isoform PKM2 contains exon 10, is abundant in proliferative cells (including cancer cells) and promotes aerobic glycolysis. In contrast, the adult isoform PKM1 contains exon 9, is highly expressed in differentiated cells, and promotes oxidative phosphorylation. Glucose metabolism is critically determined by both their absolute levels and the ratio of PKM1:PKM2. These vary among different cells and tissues and dysfunction or unbalanced expression of either isoform can cause growth arrest or even cell death [5–7].

PKM isozyme levels can change in a reciprocal manner. A gradual reduction of PKM2 in primary cells gradually induced PKM1 expression, which led to decreased nucleotide production and DNA synthesis, and proliferation arrest [8]. During neuronal differentiation, pyruvate kinase gene splicing is switched from PKM2 to PKM1. The ensuing switch from aerobic glycolysis to oxidative phosphorylation ensures survival of neurons once they differentiate [9].

Different cells and tissues employ various RNA-binding enzymes for pre-PKM splicing. For example, hnRNP (heterogeneous nuclear ribonucleoprotein) A1, A2, and I (also known as PTBP1) share an hnRNP-L_PTB motif and bind repressively to sequences flanking exon 9, resulting in the inclusion of exon 10 and a higher PKM2/PKM1 ratio in human gliomas [10], brain tumor cells [11], and pancreatic ductal adenocarcinoma cells [12]. SRSF3 (serine and arginine-rich splicing factor 3) has a different RNA recognition motif (RRM), and also cooperates with PTBP1 and hnRNPA1 to increase the PKM2/PKM1 ratio [13]. RBM4 (RNA binding motif protein 4) has two RRMs and one AIR1 (arginine methyltransferase-interacting protein) motif. RBM4 can antagonize the function of PTBP1 to induce the expression of neuronal genes and increase mitochondrial respiration capacity in mesenchymal stem cells (MSCs), thereby promoting neuronal differentiation [14].

Oocytes, unlike spermatozoa, carry the majority of extragenetic substance and factors needed for fertilization and early embryo development. As energy unbalances can severely compromise oocyte quality, i.e. decrease mitophagy, induce abnormal mitochondria aggregation, elevate radical oxygen species (ROS) production, impair cytoskeleton dynamics, and promote apoptosis, they require a tight control of energy metabolism [15–17].

Although PKM1 and PKM2 are essential for cellular energetics, their expression profile and function in mammalian oocytes have not yet been explored. In the present study, we first characterize PKM1 and PKM2 expression in mouse oocytes and assess the effects of specific knockdown of PKM isoforms on oocyte quality and viability. Then we demonstrate that KBTBD8, a member of the KBTBD (Kelch repeat and BTB domain-containing protein) family with ubiquitin ligase activity, selectively regulates PKM1 levels and its depletion also compromises oocyte quality. Finally, through transcriptome analyses and confirmatory immunoblotting experiments we identify additional members of the signaling cascade linking KBTBD8 and PKM1, and delineate a network of potential KBTBD8 interactions.

## RESULTS

### PKM1 is essential for oocyte maturation and quality

**Figure 1:**
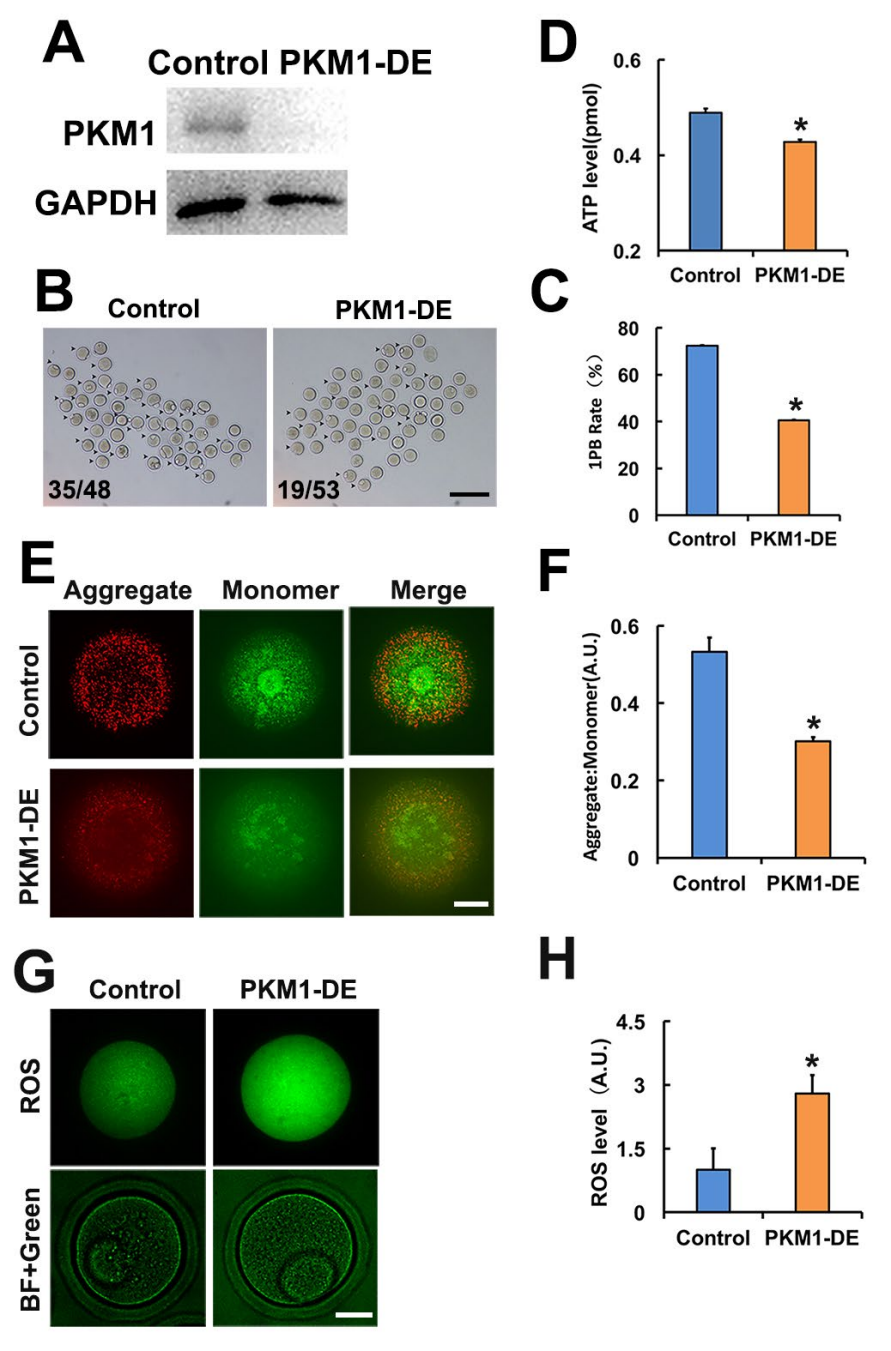
**PKM 1 is essential for oocyte maturation and quality. A.** Western blot results confirming nearly complete PKM1 depletion induced by specific antibody transfection. GAPDH was used as a loading control. **B.** Bright field images of control and PKM1-depleted (PKM1-DE) oocytes. Arrowheads indicate oocytes containing first polar bodies (1PB). Numbers indicate the ratio of 1PB oocytes/total oocytes. **C.** Quantification of 1PB presence in control and PKM1-depleted oocytes. **D.** ATP quantification in control and PKM1-depleted oocytes. **E.** JC1 fluorescence indicative of decreased mitochondrial membrane potential after PKM1 depletion. JC1 aggregates are red, monomers are green. **F.** Quantification of JC1 aggregate/monomer ratio in control and PKM1-depleted oocytes. **G.** ROS generation, revealed by DCFH-DA staining (green fluorescence), in control and PKM1-depleted oocytes. BF: Bright field. **H.** Quantification of ROS levels in control and PKM1-depleted oocytes. Scale bar for B, 200 µm; for E and G, 20 µm. * p ˂ 0.05.

Studies addressing PKM1/PKM2 functions in oocytes are lacking. Attending concerns about incomplete depletion of target protein after siRNA-mediated knockdown, and based on the successful use of antibody-mediated protein depletion [18] and our previous experience in oocytes [19], we decided to use antibody transfection to deplete PKM1 (Figure 1A) and other proteins of interest. PKM1 depletion significantly reduced both first polar body (1PB) extrusion (Figures 1B and 1C), and ATP content (Figure 1D). Furthermore, mitochondrial membrane potential (assessed by JC-1 staining) was also reduced (Figures 1E and 1F), while ROS levels increased significantly (Figures 1G and 1H). These results indicated that PKM1 expression crucially affects multiple aspects of oocyte quality. However, unexpectedly, PKM2 depletion caused very similar effects (Supplementary Figures 1A and 1G).

### KBTBD8 regulates PKM1 levels and is essential for oocyte quality

**Figure 2:**
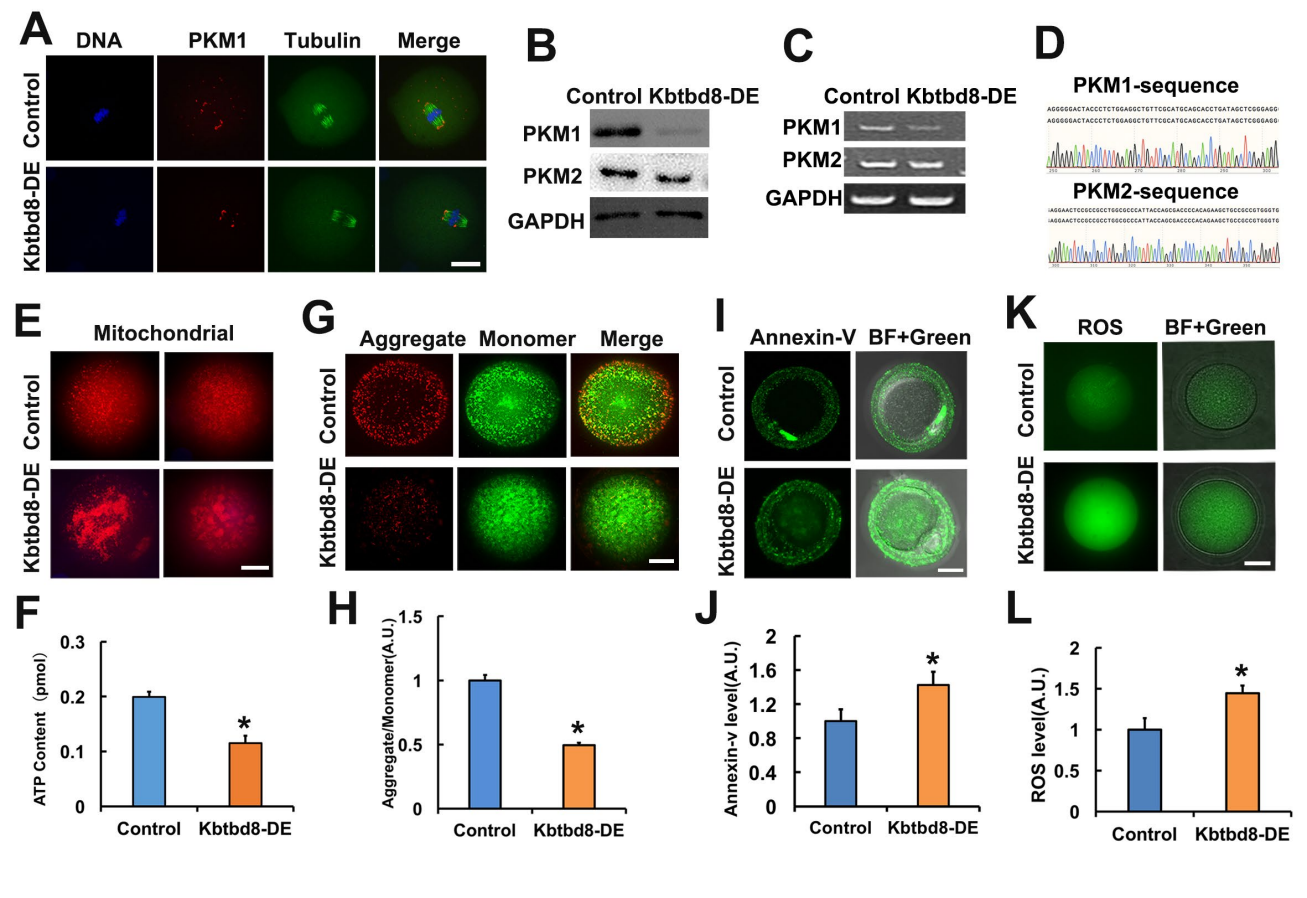
**KBTBD8 regulates PKM1 levels and is essential for oocyte quality. A.** Representative images of PKM1 and tubulin immunofluorescence in control and KBTBD8-depleted oocytes. Both cytoplasmic and spindle pole PKM1 signals decreased after KBTBD8-depletion. **B.** PKM1 and PKM2 expression in control and KBTBD8-depleted (KBTBD8-DE) oocytes. GAPDH was used as a loading control. **C.** RT-PCR assessment of PKM1 and PKM2 mRNAs. GAPDH was used as control. **D.** DNA sequencing verified that the bands in (C) are PKM1 and PKM2. **E.** Mitochondrial staining (MitoTracker Red) in control and KBTBD8-depleted oocytes. **F.** Cytoplasmic ATP contents in control and KBTBD8-depleted oocytes. **G.** Mitochondrial membrane potential (JC1 staining) in control and KBTBD8-depleted oocytes. **H.** Quantification of JC1 aggregate/monomer ratios. **I.** Apoptosis detection (Annexin V staining) in control and KBTBD8-depleted oocytes. Apoptosis increased significantly after KBTBD8 depletion. **J.** Quantification of Annexin V signal. **K.** ROS generation in control and KBTBD8-depleted oocytes. **L.** ROS quantification. Scale bars, 20 µm. * p ˂ 0.05.

Next, we investigated how is PKM1 expression regulated. In another project, we observed that KBTBD8, a female fertility factor with ubiquitin ligase activity, interacted with PKM. So herein we first compared PKM1 localization and abundance in control and KBTBD8-depleted oocytes. In control oocytes PKM1 immunofluorescence showed a scattered punctuated pattern throughout the cytoplasm, as well as enrichment at spindle poles. In contrast, PKM1 staining of both cytoplasm and spindle poles diminished significantly after antibody-mediated depletion of KBTBD8 (Figure 2A). Western blotting confirmed that KBTBD8 depletion dramatically reduced PKM1 expression, but had little effect on PKM2 levels (Figure 2B). Similarly, RT-PCR demonstrated that PKM1 mRNA levels decreased significantly, while PKM2 mRNA levels remained unchanged (Figure 2C), in KBTBD8-depleted oocytes. PKM1 and PKM2 mRNA bands were verified by sequencing (Figure 2D). Next, we examined whether oocyte quality was affected by KBTBD8 depletion. In KBTBD8-depleted oocytes the mitochondria formed large, irregularly distributed aggregates (Figure 2E). Accordingly, both intracellular ATP levels (Figure 2F), and mitochondrial membrane potential (Figures 2G and 2H) were reduced significantly. In addition, apoptosis (Figures 2I and 2J), and ROS generation (Figures 2K and 2L) were considerably elevated after KBTBD8 depletion. These results suggest that KBTBD8 depletion severely compromises oocyte quality, probably due to decreased PKM1.

### KBTBD8 is essential for meiosis and regulates multiple key kinases

**Figure 3:**
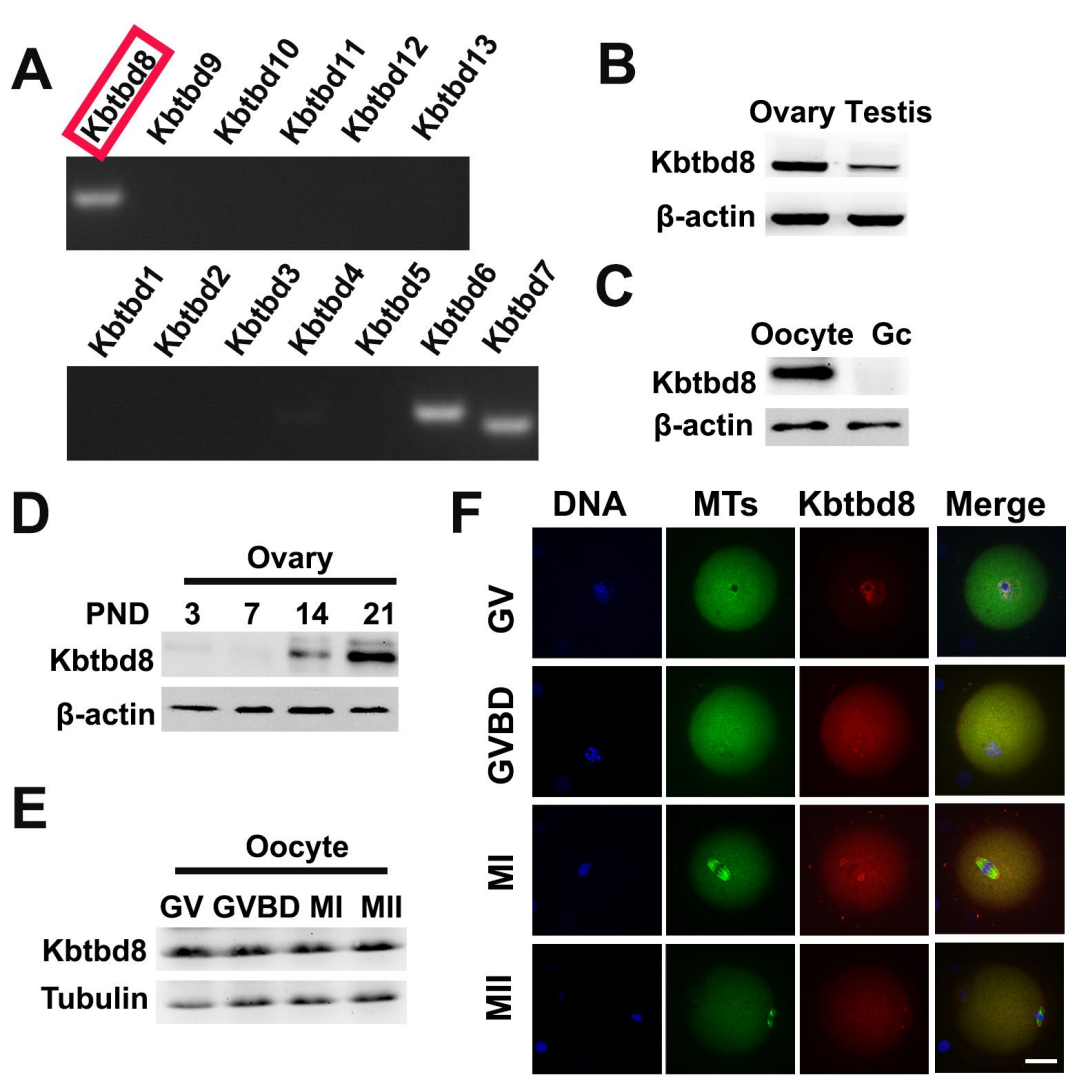
**KBTBD8 is enriched in the ovary, oocytes, and meiotic spindles. A.** Oocyte expression of KBTBD protein family mRNAs. Strong expression of KBTBD6, 7, and 8 was observed. **B.** KBTBD8 protein expression by western blot in mouse ovaries and testis. Higher KBTBD8 levels were seen in ovaries. β-actin was used as loading control. **C.** KBTBD8 is expressed in oocytes but undetectable in granular cells (Gc). β-actin was used as loading control for western blot. **D.** Chronological expression of KBTBD8 in the mouse ovary. β-actin was used as loading control for western blot. PND: post-natal day. **E.** KBTBD8 expression during oocyte meiosis. Tubulin was used as loading control for western blot. **F.** KBTBD8 immunofluorescence during meiotic stages. KBTBD8 is abundant within the nucleus at GV, enriched near chromosomes at GVBD, and concentrated at the spindle poles at MI and MII. MTs: microtubules. Scale bar, 20 µm.

Due to the significant impact of KBTBD8 inhibition on oocyte quality, we further investigated its expression during meiosis, and the effects of its depletion on meiotic phenotypes and regulatory enzymes. We first compared mRNA expression of all KBTBD family members in oocytes. KBTBD6, KBTBD7, and KBTBD8 were similarly and prominently expressed, while the other KBTBD members were almost undetectable (Figure 3A, and Supplementary Table 1). Furthermore, KBTBD8 was far more abundant in ovaries than in testes (Figure 3B), and in oocytes than in granular cells (GCs) (Figure 3C), indicating that KBTBD8 might function primarily in female gametogenesis. Temporal expression analysis showed that ovarian KBTBD8 expression was very low on post-natal day 1 (PND1), increased abruptly by PND14, and peaked on PND21, when initial follicle activation/maturation occurs (Figure 3D). These results indicated that KBTBD8 functions mainly during meiotic transition from GV to MII, rather than in the meiotic prophase (from leptone to diplotene). Next, we further examined KBTBD8 expression and subcellular localization during meiosis. Whereas KBTBD8 levels remained constant through all stages (Figure 3E), its expression was concentrated within nuclei during GV and at the spindle poles during MI and MII (Figure 3F).

**Figure 4:**
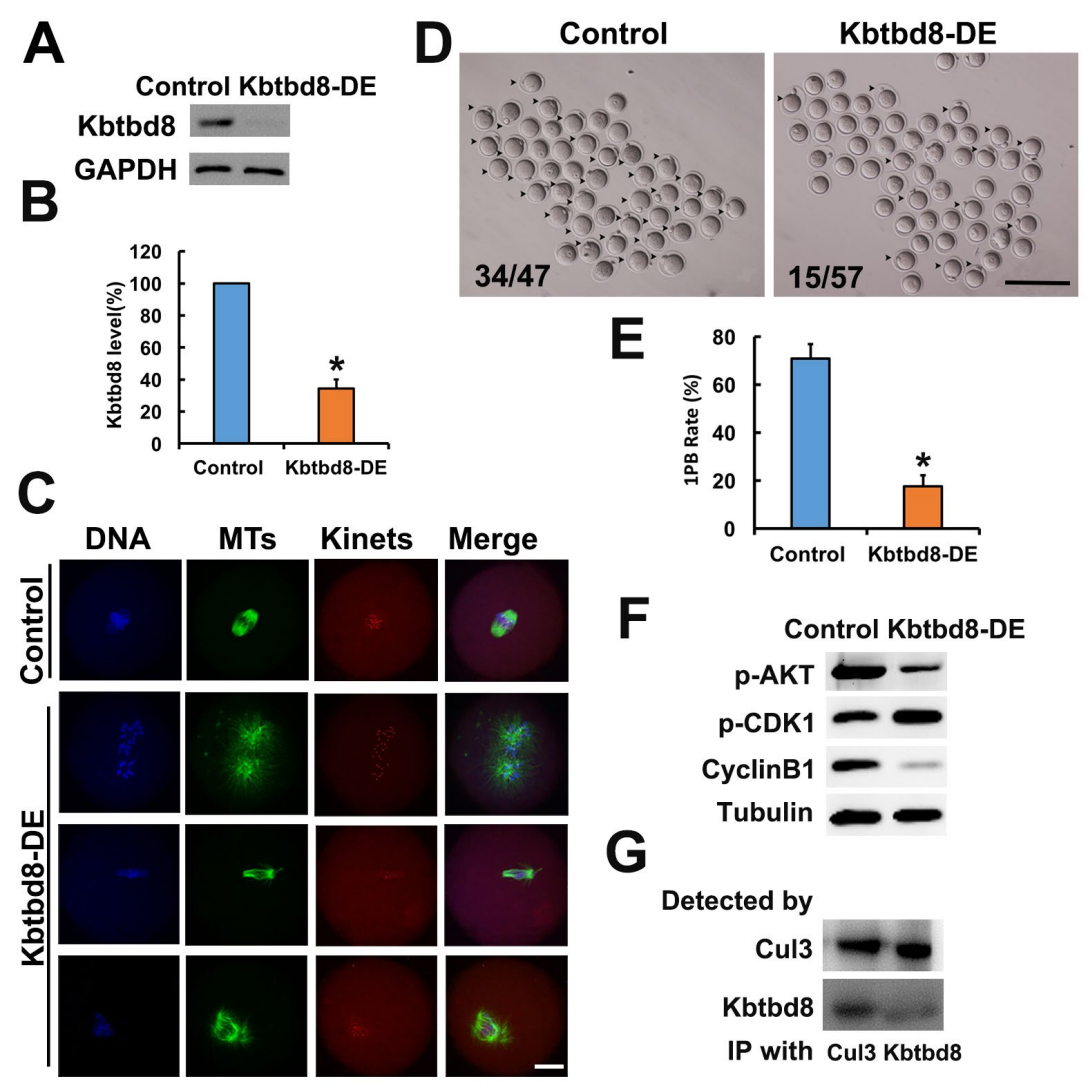
**KBTBD8 is essential for meiosis and regulates multiple key kinases. A.** Western blot confirmation of KBTBD8 depletion by specific antibody transfection in oocytes. GAPDH was used as a loading control. **B.** Quantification of KBTBD8 levels in control and KBTBD8-depleted oocytes. **C.** KBTBD8 depletion dramatically disrupted spindle organization in MI oocytes. MTs: microtubules; Kinets: kinetochores. **D.** Representative bright-field images of control and KBTBD8-depleted oocytes. Numbers in the image indicate ratio of 1PB oocytes/total oocytes. **E.** Quantification of 1PB extrusion rate. **F.** Western blots showing decreased p-Akt and cyclin B1, and increased p-Cdk1 expression in KBTBD8-depleted oocytes. Tubulin was used as a loading control. **G.** Co-IP results demonstrating that KBTBD8 interacts with cul3. Scale bars, 20 µm for C, 200 µm for D. *p ˂ 0.05.

**Figure 5:**
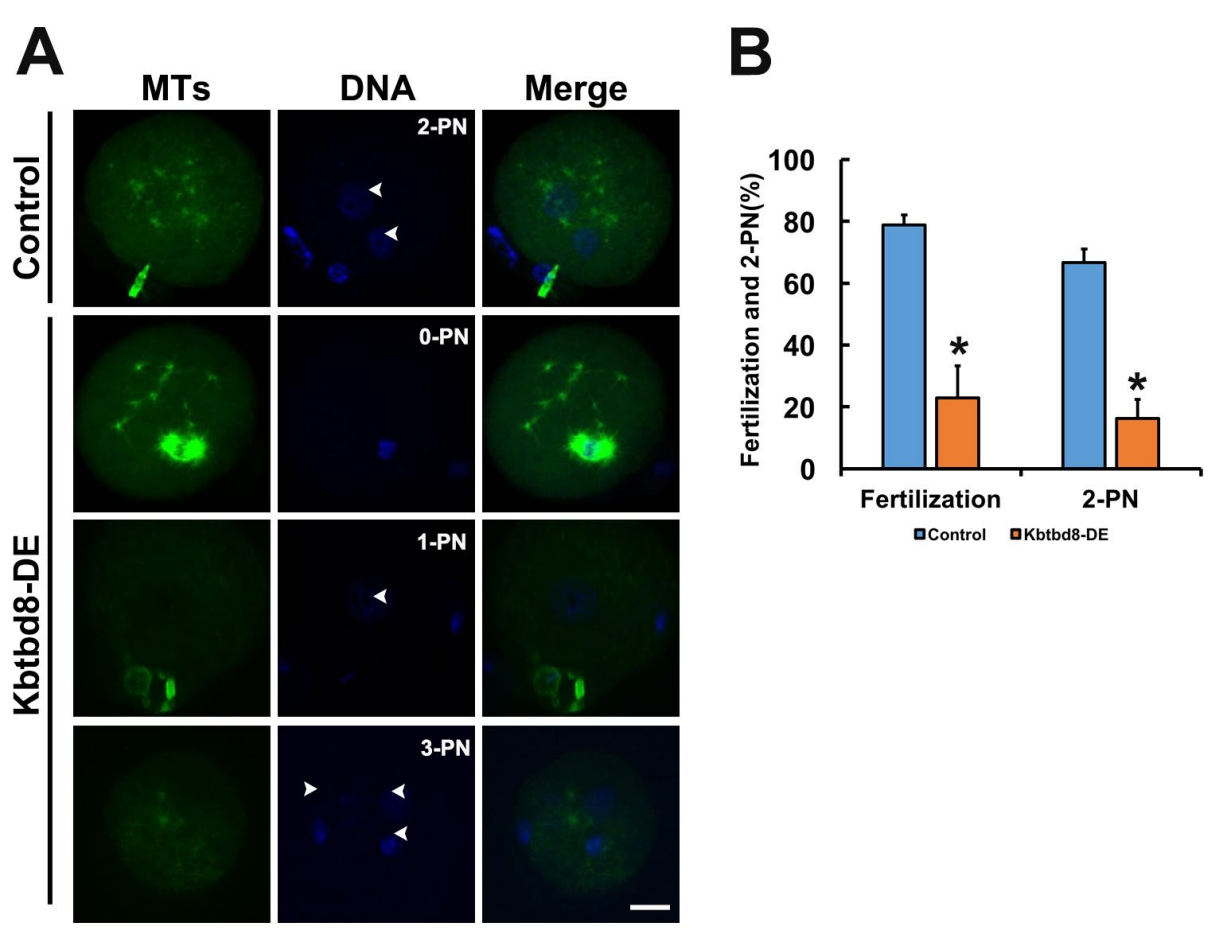
**KBTBD8 is essential for normal fertilization. A.** Immunofluorescence assessment of fertilization. Most control oocytes showed the normal 2 pronuclei (PN), while most fertilized oocytes in the KBTBD8-depleted group had abnormal pronuclei numbers (0-PN, 1-PN, or 3-PN). MTs: microtubules. Scale bar, 20 µm. **B.** Quantification of fertilization rate (fertilized oocytes/total oocytes) and 2-PN rate (2-PN oocytes/fertilized oocytes). *p ˂ 0.05.

We next examined the meiotic phenotype after KBTBD8 depletion (Figures 4A and 4B). At 8 hours of IVM (*in vitro* maturation) the spindle assembly and chromosome alignment were severely disrupted (Figure 4C); at 16 hours of IVM, the percentage of mature oocytes (as 1PB) was significantly decreased (Figures 4D and 4E). Furthermore, the fertilization rate and the rate of normally fertilized eggs (2PN) were both significantly decreased after KBTBD8 depletion (Figures 5A and 5B).

Next, we assessed whether KBTBD8 depletion affected the activities of kinases known to be essential for oocyte meiosis. We found that Cyclin B levels were reduced, while phosphorylated Cdk1 (p-Cdk1) was increased, indicating that KBTBD8 depletion decreased maturation promoting factor (MPF) activity. This notion was further supported by the finding that germinal vesicle breakdown (GVBD), which depends on MPF activity, was also decreased in KBTBD8-depleted oocytes after 3 hours of IVM (Supplementary Figures 2A and 2B). Furthermore, phosphorylated Akt (p-Akt) was also markedly diminished (Figure 4F, and Supplementary Figures 2C and 2F).

KBTBD8 has been shown to act as a ubiquitin ligase and to interact with cul3 in neurons [20]. Through CoIP experiments, we found that KBTBD8 also interacts with cul3 in oocytes (Figure 4G).

### The KBTBD8→Erk1/2→Aurora A axis regulates PKM1 levels

To uncover the mechanism by which KBTBD8 maintains oocyte quality, we initially conducted a comparative RNA-seq analysis in control and KBTBD8-depleted (by sgRNA transfection) NIH3T3 cells. We observed a string of interacting proteins from different families, where KBTBD8 and PKM seemed to be at the upstream and downstream ends, respectively, while Aurora A appeared to be in the middle. In addition, Erk1/2 could also be fitted in the cascade, upstream of Aurora A according to available references.

**Figure 6:**
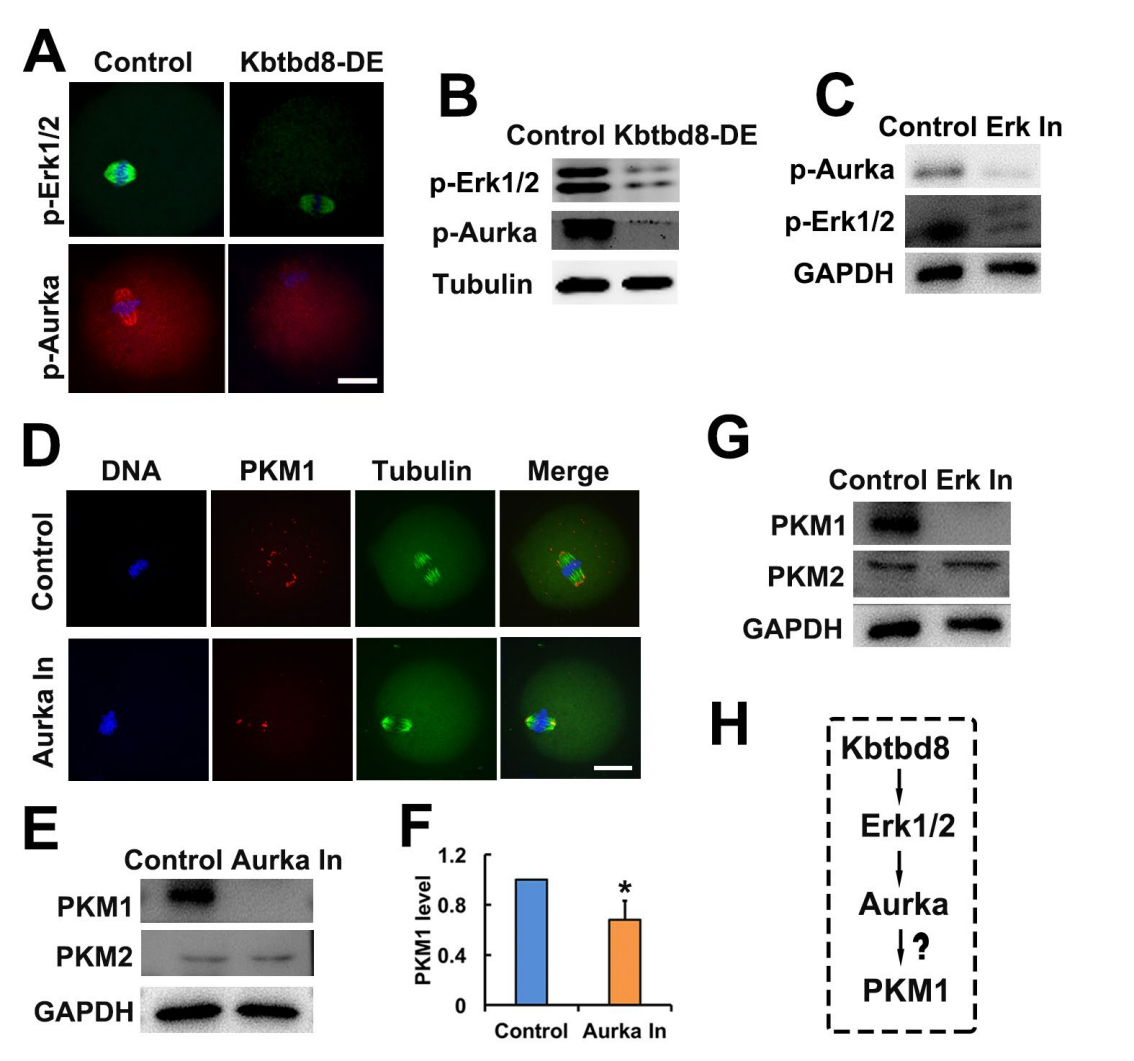
**The KBTBD8→Erk1/2→Aurora A axis regulates PKM1 levels. A.** Immunofluorescent staining of p-Erk1/2 and p-Aurora A (p-Aurka) staining in oocytes, showing decreased expression after KBTBD8 depletion. **B.** Decreased p-Erk1/2 and p-Aurora A expression after KBTBD8 depletion was confirmed by western blot. Tubulin was used as loading control. **C.** Immunoblots showing that Erk1/2 inhibition (Erk In) decreases p-Aurora A expression. GAPDH was used as loading control. **D.** PKM1 immunofluorescence showing that Aurora A inhibition (Aurka In) reduced both cytoplasmic and pole PKM1 expression. **E.** Western blot results showing that Aurora A inhibition reduced PKM1, but not PKM2, expression. GAPDH was used as loading control. **F.** Densitometric quantification of PKM1 immunoblotting data from experiments like those shown in (E). **G.** Western blots showing that Erk1/2 inhibition also reduced PKM1 expression, without affecting PKM2. **H.** Signaling pathway model of KBTBD8-mediated control of oocyte energy metabolism. Scale bar, 20 µm, *p ˂ 0.05.

Supporting this model, we found that KBTBD8 depletion led to decreases in phosphorylated Erk1/2 (p-Erk1/2) and phosphorylated Aurora A (p-Aurora A) (Figures 6A and 6B), suggesting that KBTBD8 acts upstream of these two proteins. Next, we found that Erk1/2 inhibition decreased p-Aurora A levels (Figure 6C), confirming that Aurora A is a downstream target of Erk1/2. Therefore, the final integrated string seemed to be KBTBD8→Erk 1/2→Aurora A→PKM.

Next, we investigated whether the kinases above regulated the expression of PKM isoforms. We found that similar to KBTBD8 depletion, inhibition of either Erk1/2 or Aurora A decreased PKM1 but not PKM2 expression (Figures 6D-G). These results indicated that the KBTBD8→Erk 1/2→Aurora A pathway specifically regulates PKM1 levels (Figure 6H).

### Transcriptome-wide characterization of KBTBD8-regulated pathways

**Figure 7:**
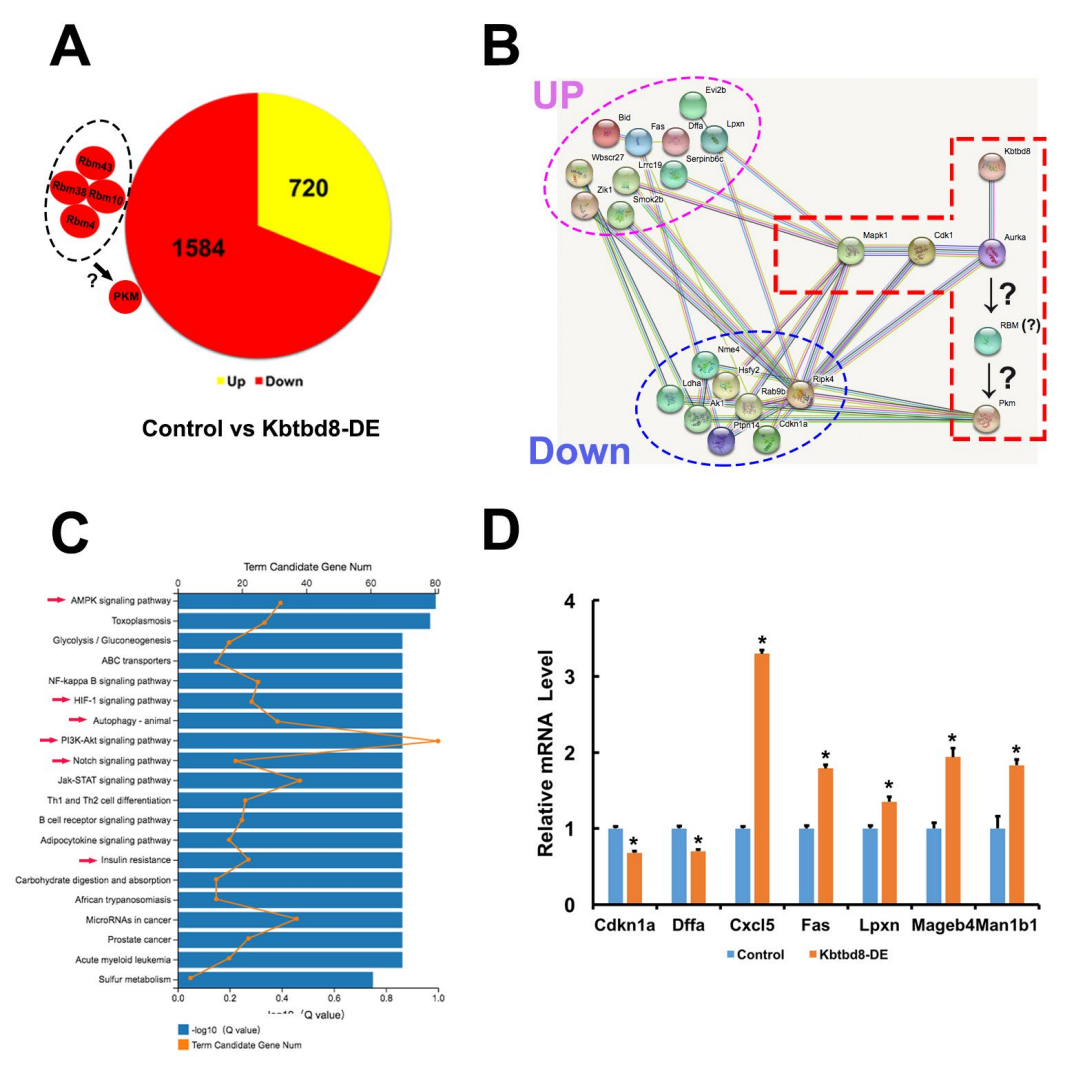
**Transcriptome-wide characterization of KBTBD8-regulated pathways. A.** Comparative mRNA sequencing in control and KBTBD8-depleted oocytes identified 2304 differentially-expressed genes (KBTBD8/control, log2 > 2). Among these, 1584 (69%) were downregulated while 720 (31%) were upregulated. Although the PKM log2 ratio was lower than 2 (90.83/50.51 = 1.84), its downregulation is consistent with our immunofluorescence and molecular analyses in KBTBD8-depleted oocytes. Also, the log2 ratios of 3 potential splicing enzymes for PKM1, RBM10, 38, and 43, were over or close to 2, so they were also considered as downregulated. Although the log2 ratio of RBM4 was only 0.73, based on its reported splicing activity on PKM1 we included it as another potential PKM1 splicing factor. **B.** Protein network showing the 18/200 top differentially expressed genes (log2 ratio > 5) that interact with the proteins in our pathway model. Of those, 10 were upregulated and 8 were downregulated. **C.** Overall pathway classification of all differentially-expressed genes. Multiple pathways, essential for the regulation of oocyte meiosis and quality, are represented; they include AMPK, HIF-1, Notch, PI3K-Akt, insulin resistance, and autophagy (red arrows). **D.** q-PCR expression analysis of 7 of the 200 differentially expressed genes verified the credibility of our sequencing data.

Next, we compared mRNA sequencing data between control and KBTBD8-depleted oocytes to further uncover whether KBTBD8 might regulate oocyte meiosis and maintain oocyte quality via other mechanisms. Overall, there were 2304 differentially expressed genes (KBTBD8/control log2>2; Supplementary Datasets 1 and 2). Of those, 69% (1584) were downregulated (Figure 7A, and Supplementary Dataset 1), indicating that the primary effect of KBTBD8 was to stabilize mRNAs. Although the log2 ratio for PKM was less than 2 (90.83/50.51=1.84), its downregulation is consistent with that seen in KBTBD8-depleted oocytes, and agrees well with our results (Figure 2A). Thus, PKM was included among other downregulated genes (Figure 7A, individual small red region).

We then searched for differentially expressed splicing proteins that may participate in PKM splicing. Of 44 RBM members, RBM10, 38, and 43 were downregulated (log2 ratios ≈2) (Supplementary Dataset 3). RBM4 has been reported to increase the PKM1/PKM2 ratio during the differentiation of MSCs into neurons [14]. Although the log2 ratio of RBM4 was just 0.73, (Supplementary Dataset 3), changes at the mRNA level do not always reflect those occurring at the protein level, so we included this RBM as a candidate for PKM splicing as well. Thus, we propose that these RBMs might be the splicing enzymes that convert pre-PKM into PKM1 (Figure 7A).

Next, we focused on the top 200 (log2 ratios > 5) differentially expressed genes and analyzed their interactions. We found that 18 of those genes could interact with the proteins in our pathway model (Figure 7B), suggesting that KBTBD8 might affect the stability or function of other proteins besides those in our model.

Next, we completed an overall pathway classification of all the differentially expressed genes, which revealed their involvement in multiple pathways (e.g. AMPK, HIF-1, Notch, PI3K-Akt, etc) essential to the regulation of oocyte meiosis and quality (Figure 7C and Supplementary Table 2). Finally, q-PCR analysis of 7/200 genes identified above verified the credibility of our sequencing data (Figure 7D and Supplementary Table 3). These results indicated that, in addition to the signaling pathway highlighted in the present study, KBTBD8 might also function in other pathways in oocytes.

## DISCUSSION

In the present study we demonstrated for the first time that PKM1 and PKM2 are critical to maintain normal ATP levels in oocytes, and identified an enzymatic cascade (KBTBD8→Erk 1/2→Aurora A) that selectively regulates PKM1 levels without affecting PKM2. These findings shed light about the mechanisms by which oocytes maintain energy metabolism. We demonstrated that an oocyte-enriched ubiquitin ligase, KBTBD8, part of the KBTBD family, affects cellular levels of important kinases (Aurora A, Cdk1, Akt, and Erk 1/2). However, our most intriguing and novel observation is that KBTBD8 regulates the expression of PKM1. Optimal PKM1 levels are essential for normal metabolism of differentiated cells, among which oocytes constitute a particular type. Accordingly, PKM1 depletion decreased ATP levels, depolarized mitochondria, increased ROS generation, and promoted apoptosis, dramatically decreasing oocyte quality. However, the mechanisms controlling the energy balance of oocytes appear to be more complex: first, both PKM1 and PKM2 are present in oocytes; second, KBTBD8 depletion only decreased PKM1, while PKM2 levels remained unchanged; third, PKM2 inhibition also damaged oocyte meiosis and quality. Although the GV oocyte is a highly differentiated cell, it could still perform two rounds of divisions to produce four cells in a short time (within a day); therefore, aerobic glycolysis by PKM2 might be more efficient for this purpose. Alternatively, other factors might control whether the PKM pre-mRNA is spliced into PKM2, so further investigation is warranted.

Different members of the same protein family commonly interact with diverse upstream regulators and downstream effectors, and may have both redundant and unique functions. KBTBD family members reflect well this reality. For example, several KBTBD members form complexes with ubiquitin ligase and promote the ubiquitination and subsequent degradation of substrates [21–26]. However, they also interact with several other partners affecting diverse cellular processes. For example, both KBTBD1 (Klhl31) and KBTBD10 (Klhl41) depletion cause nemaline myopathy, although the mechanisms are different: while KLHL31 targets Filamin C, a muscle-specific actin-crosslinking protein, for ubiquitination and degradation [23], KLHL41 is poly-ubiquitinated and prevents aggregation and degradation of nebulin, an essential component of the sarcomere. Thus, inhibition of KLHL41 poly-ubiquitination prevents its stabilizing effect on nebulin [27]. A previous study demonstrated that CUL3-KBTBD8 promotes RNA polymerization by ubiquitylating NOLC1-TCOF1 in neural crest cells [20]. In the oocytes of the present study, KBTBD8 appears to promote Erk 1/2→Aurora A signaling to ensure optimal levels of PKM1. Therefore, KBTBD8 might function differently in different cells.

While our evidence links KBTBD8 and PKM1, there are no reports of KBTBD proteins with RNA-binding capacities, and neither kelch motif proteins nor BTB/POZ domain proteins have been reported to mediate RNA splicing. Therefore, there must be intermediate factors between KBTBD8 and PKM. Based on our own and others’ data, we proposed the KBTBD8→Erk1/2→Aurora A axis as the core protein string therein, and conducted a series of experiments to evaluate this model. Although none of the KBTBD family proteins have been shown to regulate Erk1/2 activity, available data indicated that kelch proteins have this functionality. For example, in cholangiocarcinoma cells, KLH21 depletion significantly decreased Erk 1/2 phosphorylation (activation] [28]. Second, multiple studies demonstrated that Erk1/2 regulated RNA splicing in different cells [29–31], while Aurora A was screened as a regulator in the alternative splicing of Bcl-x and Mcl1 in HeLa cells; thus, Erk 1/2→Aurora A might regulate pre-mRNA splicing of other genes in oocytes as well [32]. Third, although there is no evidence linking Erk1/2 and Aurora A activity in oocytes, Erk1/2 activity was reported to increase translational levels of Aurora A in melanoma [33].

We speculate that there must be specific splicing enzymes between Erk1/2→Aurora A and PKM1 since neither Erk1/2 nor Aurora A have RNA-binding motifs (Figure 7B), although in oocytes evidence for this is lacking. RBM4 (RNA binding motif protein 4) was reported to antagonize the function of PTBP1 and increase PKM1/PKM2 ratio during the differentiation of MSCs into neurons [14]. In our transcription-wide study, the expression of 3 out of 44 Rbm members decreased close to or more than 4-fold (Supplementary Dataset 3) after KBTBD8 depletion, so one of these might be the direct splicing factor of the PKM1 transcript (Figure 7A).

Ubiquitination complexes have many substrates, so it is possible that the pathway we determined above is incomplete. Comparison of the transcriptome profiles of control and KBTBD8-depleted oocytes revealed that 18 of the top 200 differentially expressed genes had multiple interactions with the proteins in our KBTBD8 pathway model. Although there are very few reports on their functions in female reproduction (except for some well-known apoptosis-promoting genes such as Fas and Bid, which were upregulated in KBTBD8-depleted oocytes), their known primary function in other processes suggests potential roles in maintaining oocyte quality. For example, among the 8 downregulated genes, RIPK4 (receptor-interacting serine/threonine kinase 4) promotes pancreatic cancer cell migration and invasion by activating RAF1/MEK/ERK signaling [34]; presumably, RIPK4 downregulation in oocytes may also inactivate Erk signaling and impair oocyte quality. Interestingly, RIPK4 activity could also be regulated by ubiquitin ligase [35]. Among the 10 upregulated genes, Lrrc19 (leucine rich repeat containing 19) promotes gut inflammation [36] and induces the expression of proinflammatory cytokines [37]; if active in oocytes, a similar mechanism might also impair their quality. These results suggest that KBTBD8 might control additional and essential factors not present in our model, such that multiple interactions are possible (Figure 7B).

In conclusion, our study demonstrated for the first time that the oocyte-enriched ubiquitin ligase KBTBD8 is essential for oocyte quality. The proposed mechanism can be summarized as follows: KBTBD8 regulates PKM pre-mRNA splicing through Erk 1/2→Aurora A and one or more RBMs. These findings, and additional evidence of its potential involvement in multiple other pathways, position KBTBD8 as a critical determinant of oocyte quality.

## MATERIALS AND METHODS

### Chemicals, antibodies, and inhibitors

All chemicals and reagents were obtained from Sigma (St. Louis, MO, USA) unless otherwise stated. Primary antibodies were mouse anti-β-actin (Cat#: A5316-100; Santa Cruz, Dallas, TX, USA); mouse anti-GAPDH (Cat#: 30201ES60; YEASEN, Shanghai, China); human anti-centromere CREST (Cat#: 15-234; Antibodies Incorporated, Davis, CA, USA); rabbit polyclonal anti-phospho Aurora Kinase (p-Thr288) (Cat#: SAB4503898; Sigma); rabbit polyclonal anti-cyclin B1 (Cat#: 55004-1-AP; Proteintech, Rosemont, IL, USA); rabbit polyclonal anti-Kbtbd8 (Cat#: 1096; ZoonBio Biotechnology, Nanjing, China); rabbit polyclonal anti-CDK1 (p-Thr14) (Cat#: OAAN02724; Aviva Systems Biology, San Diego, CA, USA); mouse anti-diphosphorylated Erk-1&2 (Cat#: M9692; Sigma); rabbit polyclonal anti-PKM1 (Cat#: ab116271; Abcam, Cambridge, UK); and rabbit polyclonal anti-PKM2 (Cat#: BS6443; Bioworld Technology, Dublin, OH, USA). Secondary antibodies were Cy2-conjugated donkey anti-mouse IgG (Code: 715-225-150), Rhodamine (TRITC)-conjugated donkey anti-human IgG (Code: 709-025-149), Cy2-conjugated donkey anti-human IgG (Code: 709-225-149), and Cy2-conjugated donkey anti-rabbit IgG (Code: 711-225-152), all purchased from Jackson ImmunoResearch Laboratory (West Grove, PA, USA). Horseradish peroxidase (HRP)-conjugated rabbit anti-goat IgG and HRP-conjugated goat anti-mouse IgG were purchased from Vazyme (Nanjing, Jiangsu, China). Aurora A Inhibitor I (Cat#: S1451) and the Erk1/2 inhibitor SCH772984 (Cat#: S7101), both from Selleck Chemicals (Houston, TX, USA), were dissolved in dimethyl sulfoxide at a concentration of 5 or 10 mM. GV oocytes were treated with 10 μM Aurora A Inhibitor or ERK1/2 inhibitor for 24 h.

### Oocyte collection and culture

GV oocytes were obtained from the ovaries of 3-4 weeks-old ICR mice supplied by the Animal Core Facility of Nanjing Medical University. The mice were euthanized by CO_2_ inhalation and cervical dislocation, and ovaries were isolated and placed in operation medium (Hepes) with 2.5 nM milrinone and 10% fetal bovine serum (FBS) (Gibco, Waltham, MA, USA). Oocytes were removed from the ovary by puncturing the follicles with a hypodermic needle. Cumulus cells were washed off the cumulus-oocyte complexes, and groups of 50 isolated denuded oocytes were placed in 100 μl droplets of culture medium under mineral oil in plastic dishes. The culture medium was MEM+ (MEM with 0.01 mM EDTA, 0.23 mM Na-pyruvate, 0.2 mM penicillin/streptomycin, 3 mg/ml BSA) containing 20% FBS. Oocytes were grown at 37.0°C, 5% O_2_, 5% CO_2_ in a humidified atmosphere. Before in vitro maturation (IVM), all culture media included 2.5 nM milrinone to prevent the resumption of meiosis. All experiments were approved by the Animal Care and Use Committee of Nanjing Medical University and were performed following institutional guidelines.

### Antibody transfection

Chariot™ Protein Delivery Reagent (Active Motif, Carlsbad, CA, USA) was used for antibody transfection. Briefly, two tubes, one containing 1 µl Chariot reagent (1mg/ml in 50% DMSO) in 5 µl sterile water, and the other 1 µg antibody in PBS (final volume = 6 µl) were first set up. Next, both solutions were mixed together gently and incubated at room temperature for 30 min to allow the formation of Chariot-IgG complexes, which were then added into the 100 µl MEM+ droplet containing 50 oocytes. After 12–14 h, the oocytes were washed to remove the complex-containing MEM+, and after 1–2 h another two rounds of antibody transfection were repeated to ensure the effectiveness of antibody-mediated inhibition. During the whole procedure, typically 40–44 h long, 2.5 nM milrinone was always included to prevent the resumption of meiosis. Next, oocytes were transferred into milrinone-free MEM+ and cultured for 8 or 16 h, then subjected to the experiments described below. Antibodies for transfection were thoroughly buffer-exchanged (over 10^4^ dilutions of original buffer) into PBS/50% glycerol with size-exclusion spin columns (Millipore, cutoff, 100KDa; spin speed, 5000 rpm) to remove antiseptics (usually NaN3) contained in the original formulation.

### Mitochondrial staining and ATP measurements

For mitochondrial staining, oocytes were incubated in Hepes containing 100 nM Mito Tracker (Cat#: M7521, Invitrogen, Carlsbad, CA, USA) and 10 µg/ml Hoechst 33342 (Sigma) for 30 min. Images were taken with an Andor Revolution spin disk confocal workstation.

For ATP measurements, the oocytes were first lysed with 100 µl ATP lysis solution (Cat#: S0026, Beyotime) on ice. The samples were then detected by enzyme-labeled instrument Synergy2 (BioTek, Winooski, VT, USA) to evaluate ATP level.

### Assay of mitochondrial transmembrane potential

Oocytes were incubated at 37°C for 20 min with the fluorescent potentiometric indicator JC-1 (Cat#: 40706ES60, YEASEN) diluted 1:200, then washed twice with PBS and added to droplets (50 µl) of culture medium. Images of green fluorescence (JC-1 as monomers at low membrane potentials) and red fluorescence (JC-1 as aggregates at higher membrane potentials) were captured using confocal microscopy as above. Mitochondrial depolarization is indicated by a decrease in the red/green fluorescence intensity ratio.

### Detection of ROS generation

The ROS Assay Kit (Cat#: S0033, Beyotime) was used to detect ROS generation in oocytes. Briefly, oocytes were incubated with dichlorofluorescein diacetate (DCFH-DA) probe for 20 min at 37°C in the dark, washed, and mounted on slides for confocal imaging.

### Apoptosis detection

Apoptosis was detected with an Annexin V-FITC/PI Apoptosis Detection Kit (Cat#: 40302ES20, YEASEN). Oocytes were stained with 5 μl annexin V‐FITC and 10 μL PI Staining Solution diluted in 100 μl binding buffer for 15 min in the dark at RT. After washing, oocytes were mounted onto glass slides, and images were obtained as above.

### Immunofluorescence staining

Oocytes were briefly washed in PBS with 0.05% polyvinylpyrrolidone (PVP), permeabilized in 0.5% Triton X-100/PHEM (60 mM PIPES, 25 mM Hepes, pH 6.9, 10 mM EGTA, 8 mM MgSO_4_) for 5 min and washed three times rapidly in PBS/PVP. Next, the oocytes were fixed in 3.7% paraformaldehyde (PFA)/PHEM for 20 min, washed three times (10 min each) in PBS/PVP, and blocked with blocking buffer (1% BSA/PHEM with 100 mM glycine) at RT for 1 h. Oocytes were then incubated at 4°C overnight with primary antibody diluted in blocking buffer, washed three times (10 min each) in PBS with 0.05% Tween-20 (PBST), incubated at room temperature for 45 min with secondary antibody diluted in blocking buffer (1:750 in all cases), and washed three times (10 min each) in PBST. Finally, DNA was stained with 3 µg/ml Hoechst 33258, and the oocytes were mounted onto a slide with mounting medium (0.5% propyl gallate, 0.1M Tris-HCl, pH 7.4, 88% glycerol) and covered with a cover glass (0.13–0.17 µm thick). To maintain oocytes’ structure, two strips of double-stick tape (90 µm thick) were positioned between the slide and cover glass. Images were obtained as above.

### Co-Immunoprecipitation (Co-IP)

For Co-IP experiments, 5 μg of rabbit anti-KBTBD8 or rabbit anti-Cul3 antibody was first coupled to 30 μl protein-A/G beads (Macgene, Beijing, China) for 4 h at 4 °C on a rotating wheel in 250 μl IP buffer (20 mM Tris-HCl, pH 8.0, 10 mM EDTA, 1 mM EGTA, 150 mM NaCl, 0.05% Triton X-100, 0.05% NP-40, 1 mM phenylmethylsulfonyl fluoride) with 1:100 protease inhibitor (Sigma) and 1:500 phosphatase inhibitor (Sigma). Meanwhile, 600 oocytes were lysed and ultra-sonicated in 250 µl IP buffer and then pre-cleaned with 30 µl protein A/G beads for 4 h at 4 °C. Then, protein A/G-coupled KBTBD8 or Cul3 antibody was incubated overnight at 4 °C with pre-cleaned oocyte lysate supernatant. Finally, after three washes (10 min each with 250 ul IP buffer), the beads with bound immune complexes were subjected to western blotting with KBTBD8 or Cul3 antibody in parallel.

### RNA-sequencing

RNA sequencing was performed by BGI-Wuhan Co. Ltd. in 3 control and 3 KBTBD8-depleted oocyte batches (each containing 200 oocytes) that were freshly frozen in liquid nitrogen immediately at the end of treatment. RNA was amplified using the SMARTer Ultra Low Input RNA Kit, the resulting cDNA fragmented using the Agilent2100 chip, and paired-end libraries constructed using the Nextera XT DNA Library Preparation Kit. The libraries were sequenced on an Illumina HiSeq.

### Data analysis and statistics

All experiments were repeated at least three times. Measurements on confocal images were performed with ImageJ (NIH). Data are presented as mean ± SEM or percentages. Statistical comparisons between two groups were made with Student’s t test. Statistical significance was set at P < 0.05.

## SUPPLEMENTARY MATERIAL

Supplementary Tables and Figures

Supplementary Dataset 1

Supplementary Dataset 2

Supplementary Dataset 3
