# Supplementary material for: The ubiquitin ligase KBTBD8 regulates PKM1 levels via Erk1/2 and Aurora A to ensure oocyte quality
Source: Aging (Albany NY). 2019 Feb 20;11(4):1110–28. doi: 10.18632/aging.101802 (PMC6402520; doi:10.18632/aging.101802)
Supplement: Supplementary Tables and Figures [file aging-11-101802-s001.pdf]

SUPPLEMENTARY MATERIAL

Please browse the links in Full Text version of this manuscript to see Supplementary Datasets.

Supplementary Dataset 1. This table lists all downregulated genes (|log2| > 2) with multiple items of gene information.
Supplementary Dataset 2. This table lists all upregulated genes (|log2| > 2) with multiple items of gene information.
Supplementary Dataset 3. This table lists all RBM genes (downregulated and upregulated) without |log2| limitation, and with multiple items of gene information. Information for RBM4, 10, 38, and 43 is red-typed and yellow-highlighted.

Supplementary Tables

Supplementary Table 1. RT-PCR primers for mouse KBTBD family members.

Name		Sequence	Length
Kbtbd1	Forward	5'-TCATTTCTGCTGCTGTGTACC	293bp
	Reverse	5'-CCATTTCATTGCGATCTGG	
Kbtbd2	Forward	5'-GCAGAGTGCAAAGAGGATGG	296bp
	Reverse	5'-CCTGAACCACAACAGACTTATCG	
Kbtbd3	Forward	5'-CAGTTGTCATCCTTTCTTCAGG	293bp
	Reverse	5'-CTTAATCCATGTGAGGACAGC	
Kbtbd4	Forward	5'-GAGTGTTCTCGGTTTCTGG	299bp
	Reverse	5'-CCTTCAAGCTAGTCCTTAGTGAC	
Kbtbd5	Forward	5'-AATTGCCTGGCTGTCTTCC	281bp
	Reverse	5'-CGAACGCTCTCGAAGACC	
Kbtbd6	Forward	5'-GGTGGACTACTGCTACACTGG	318bp
	Reverse	5'-AAGCTGTCCAGACGCAGG	
Kbtbd7	Forward	5'-ATGCTGCAGCTGGAGTACG	289bp
	Reverse	5'-ACTGCACCGCCACATGAC	
Kbtbd8	Forward	5'-CATCAGGAGCTTGGAGATCG	328bp
	Reverse	5'-CACTGGTGTTGGAGGGTTC	
Kbtbd9	Forward	5'-CAGTATCCACTTTGCCTGG	291bp
	Reverse	5'-GGTCTGTAAATGCTTTCGCTC	
Kbtbd10	Forward	5'-TAGACAACGTGGATCCTGC	296bp
	Reverse	5'-CAGTTGCATGAAATCTTCTTCC	
Kbtbd11	Forward	5'-CTGCTACGAGGTGCTGAG	291bp
	Reverse	5'-GCCTGGTAGCAATAAACG	
Kbtbd12	Forward	5'-ACATCGAAGCTCACCAGC	297bp
	Reverse	5'-CATAGCGAAGGTTGGAGG	
Kbtbd13	Forward	5'-AGGACGAGTTGCTGCAGG	299bp
	Reverse	5'-CGACGCATCCTCTAGGAAGC	

These primers were used in Figure 3A.

Supplementary Table 2. KEGG pathway classification of all differentially-expressed genes in KBTBD8-depleted oocytes.

KEGG pathway	Name of Genes
PI3K-Akt signaling pathway*	Gm2446,Ccdc149,Gm31532,Gm38469,Pik3r6,Raf1,Akt1,Bcl2,Bcl2l1,Ccnd2,Ccne1,Cdkn1a, Col1a1, Creb1,Csf1,Efna4,Eif4ebp1,Fgf8,Fgfr1,Flt1,Il2rb,Il3,Irs1,Itgb6,Lama1,Lama3,Lbp,Sgk3, Mtcp1,Nfkb1,Ocm,Osmr,Pdgfb,Pik3r2,Itgb4,Rela,Sos2,Spp1,Creb3l2,Wrap53,Eif4e1b,Thbs4,Tnr, Tsc2,Brat1,Spon1,C1qtnf5,Ppp2r3a,Elmsan1,Tenm3,Rgs9bp,Plekhg3,Angptl2,Ppp2r2c,Ccbe1, Tnn,Colq,Ppp2r1a,Lsr,Pdgfc,Prl2a1,Fgf21,Mtor,Frmpd1,Gm14025,G6pc3,Pdgfd,Angptl1,Ddit4l, Crtc2,Rptor,Pck2,Ddit4,Pik3ap1
AMPK signaling pathway*	Gm2446,Ccdc149,Acaca,Akt1,Cpt1a,Creb1,Eif4ebp1,Irs1,Pfkl,Pik3r2,Ppargc1a,Creb3l2, Tsc2,4931409K22Rik,Ppp2r3a,Elmsan1,Plekhg3,Map3k7,Ppp2r2c,Scd3,Ppp2r1a,Lsr,Mtor,Nsmf, Akt1s1,G6pc3,Osgin1,Crtc2,Rptor,Pck2
HIF-1 signaling pathway*	Camk2d,Akt1,Bcl2,Cdkn1a,Eif4ebp1,Eno1,Flt1Hk2,Ifngr2,Nfkb1,Pik3r2,Plcg1,Rela,Slc2a1, Stat3,Eif4e1b,Atxn2l,Angptl2,Ep300,Mtor,Angptl1
Notch signaling pathway*	Maml1,Ctbp1,Dvl2,Aes,Notch1,Kat2b,Rfng,Sned1,Os9,Ccdc148,Atxn2l,Elmsan1,Ep300, Ncstn,Dtx2,Mxra8
Autophagy signaling pathway*	Raf1,Akt1,Bcl2,Bcl2l1,Dapk3,Irs1,Mtmr4,Pik3r2,Tsc2,Def8,Rragb,Plekhg3,Map3k7, Ankar,Bend6,Plekhm1,Atg13,Rragd,Mtor,Nsmf,Gabarapl1,Atg4a,Wdr45b,Akt1s1,Ddit4l, Atg16l2,Rptor,Ddit4,Mtmr14
Insulin resistance*	Pygl,Akt1,Cpt1a,Creb1,Irs1,Nfkb1,Pik3r2,Prkce,Ppargc1a,Rela,Rps6ka2,Slc2a1,Stat3, Creb3l2,Nr1h2,Ppp1r3b,Tbl2,Mtor,G6pc3,Crtc2,Pck2

*Only pathways known to be important for oocyte quality were selected.

Supplementary Table 3. Q-PCR primers of genes with reported reproductive function.

Name		Sequence	Length
Dffa[1]	Forward	5'-AGGACGGGACCATAGTGG-3'	302bp
	Reverse	5'-ACAGCTTTGGCAGAGTTCC-3'	
Cdkn1a[1]	Forward	5'-GTGGACAGTGAGCAGTTGC-3'	282bp
	Reverse	5'-CAGAGTGCAAGACAGCGAC-3'	
	Reverse	5'-ACAGTGATTGCTGCTCTGTTG-3'	
Lpxn[1]	Forward	5'-AGGCTGGGAGCGTCTATCTG-3'	271bp
	Reverse	5'-GAGGTTTTGGGAGGCAGGAC-3'	
Fas[1]	Forward	5'-GCTTGCTGGCTCACAGTTAAG-3'	285bp
	Reverse	5'-CCTGCAATTTCCGTTTGGCT-3'	
	Reverse	5'-ACATCCAAGACTGGGGTGAG-3'	
Cxcl5[1]	Forward	5'-GTTCCATCTCGCCATTCATGC-3'	215bp
	Reverse	5'-TATGACTTCCACCGTAGGGC-3'	
Man1b1[1]	Forward	5'-GGGTGTGGATGCCTTCGTTA-3'	248bp
	Reverse	5'-TTCCGCTGTAGCCTCGAAAG-3'	
Mageb4[1]	Forward	5'-CAGAACGGGCTTTTGATGCC-3'	201bp
	Reverse	5'-ACTGTTGGCCTCCTTTTGGT-3'	
	Reverse	5'-TGGACTCATCATCTTTGCTGGT-3'	
PKM1[2]	Forward	5'-CATCAGCAAAATCGAGAACC-3'	389bp
	Reverse	5'-CTTCAAACAGCAGACGGTGG-3'	
PKm2[2]	Forward	5'-GTTTGATGAGATCTTGGAGG-3'	461bp
	Reverse	5'-TGGTGAGCACGATAATGG-3'	

[1] These 7 genes correspond to genes verified by Q-PCR in Figure 7D.

[2] PKM1 & PKM2 primers were used in Figure 2C and 2D.

Supplementary Figures

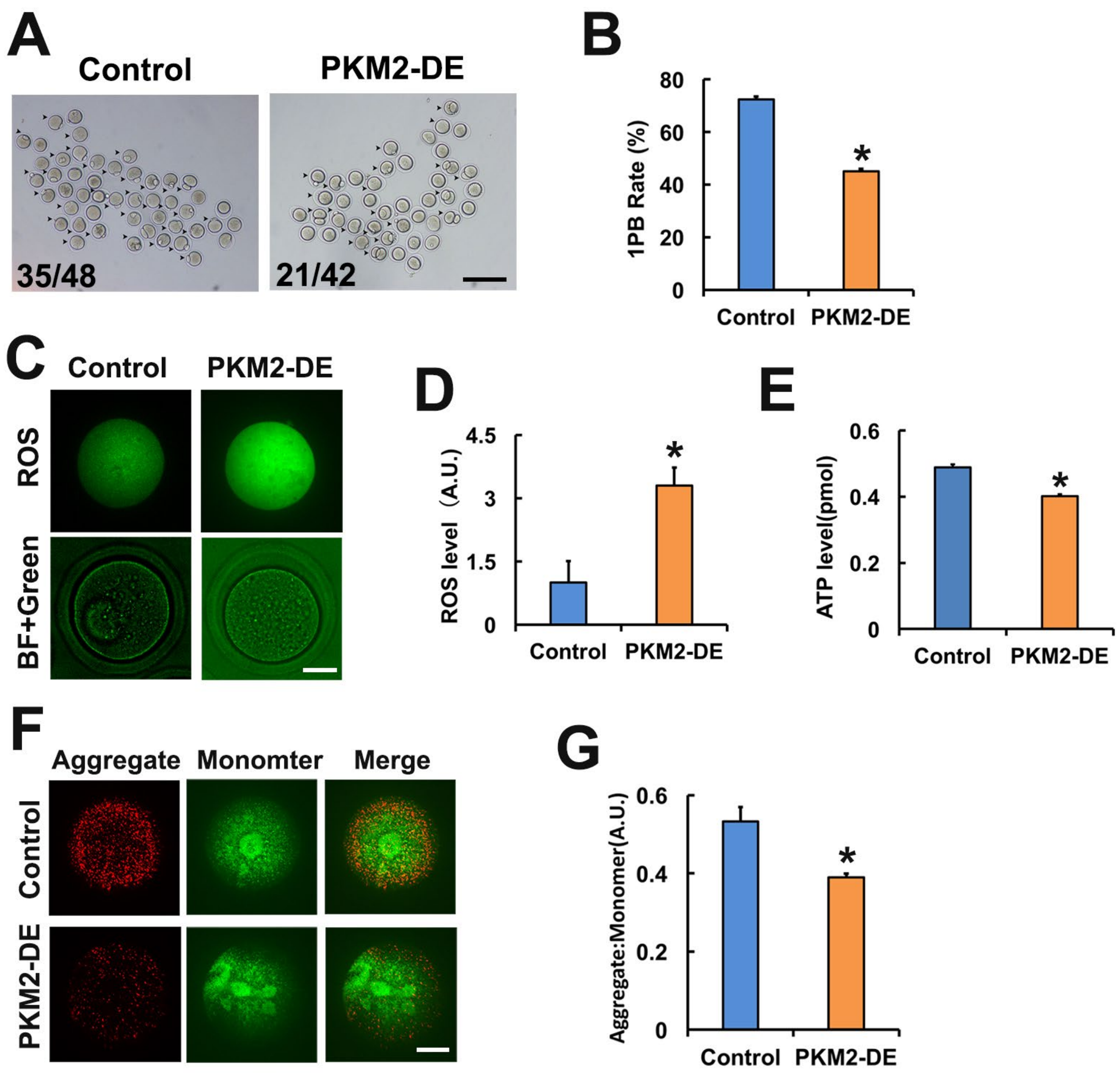

Supplementary Figure 1. PKM 2 is also essential for oocyte maturation and quality. A. Percentage of oocytes with first polar body (1PB) significantly decreased after PKM2 depletion (PKM2-DE). The number in the image represents the number of 1PB oocytes/number of total oocytes. **B.** Quantification of 1PB rate in control and PKM2-depleted groups. **C.** ROS (green) level significantly increased after PKM2 depletion. **D.** Quantification of ROS level in control and PKM2-depleted groups. **E.** Quantification showed that ATP level significantly decreased after PKM2 depletion. **F.** JC1 staining showed that mitochondria membrane potential significantly decreased after PKM2 depletion. The aggregate in red, the monomer in green. **G.** Quantification of aggregate/monomer ratio in control and PKM2-depleted groups. Scale bar for A, 200 µm; for D and F, 20µm. * indicates $p < 0.05$.

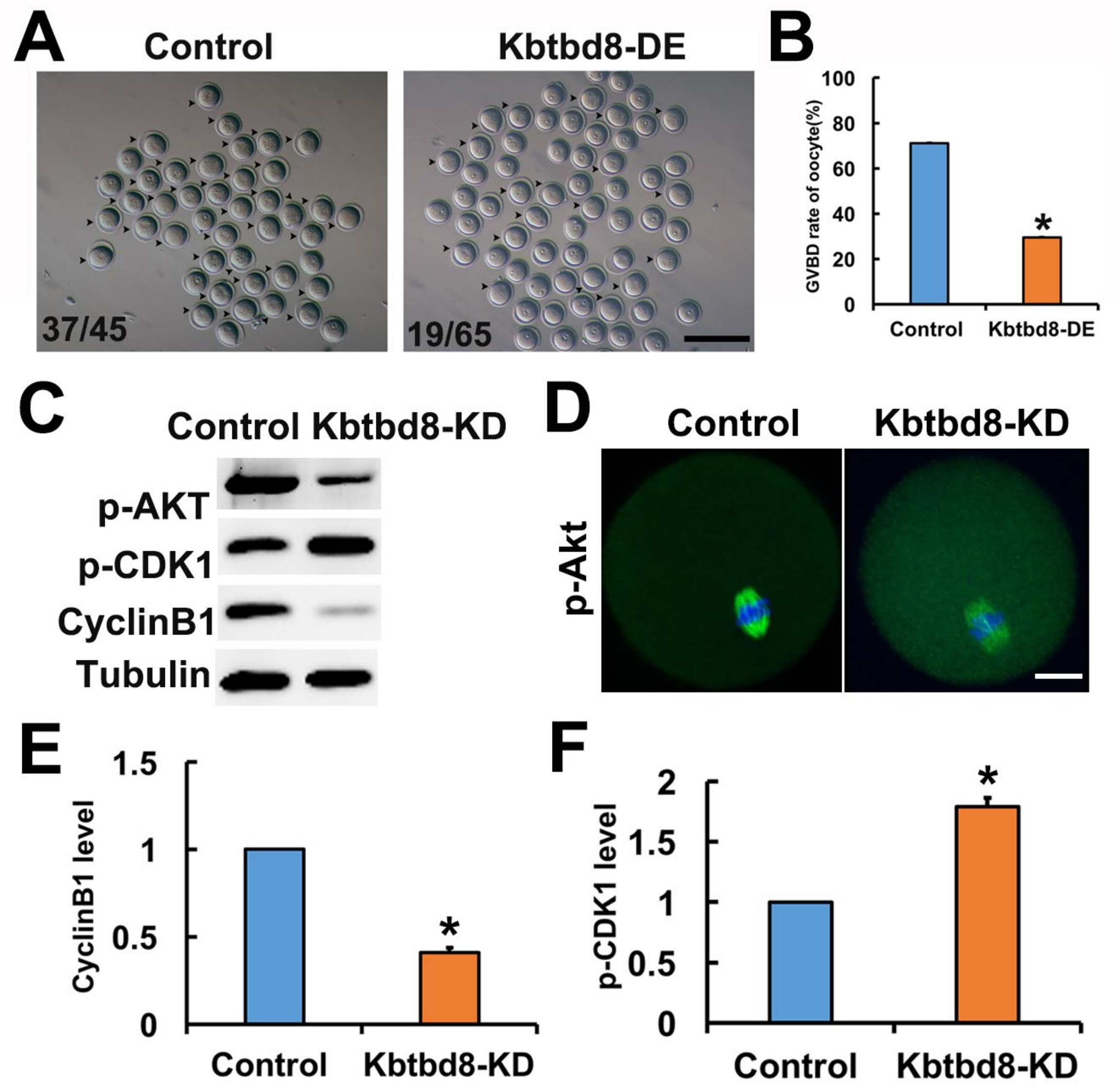

Supplementary Figure 2. KBTBD8 is also important for the activation of Akt and MPF in oocytes. **A.** KBTBD8 depletion significantly reduced the percentage of GVBD after 3 hours of IVM. Numbers in the panel are numbers of GVBD/numbers of total IVM oocytes. **B.** Quantification of the percentage of GVBD after 3 hours of IVM in control and KBTBD8-depleted groups. **C.** KBTBD8 depletion significantly reduced the p-Akt level. KBTBD8 depletion also significantly decreased MPF activity (reduced cyclinB1 level and increased p-Cdk1 level). **D.** Representative immunofluorescence images showed that p-Akt staining within spindles dramatically diminished in KBTBD8-depleted oocytes. p-Akt in green, DNA in blue. **E.** Quantification of cyclinB1 level in control and KBTBD8-depleted groups. **F.** Quantification of p-Cdk1 level in control and KBTBD8-depleted groups. Scale bar for A, 200μm; for D, 20 μm. * indicates $p < 0.05$.
